# Delayed-Onset Transient Light Sensitivity Syndrome after Corneal Collagen Cross-Linking: A Case Series

**Published:** 2019

**Authors:** Majid Moshirfar, Uma Vaidyanathan, Grant C. Hopping, Yasmyne C. Ronquillo, Phillip C. Hoopes

**Affiliations:** 1 John A. Moran Eye Center, Department of Ophthalmology and Visual Sciences, University of Utah School of Medicine, Salt Lake City, UT, USA; 2 Utah Lions Eye Bank, Murray, UT, USA; 3 Hoopes Durrie Rivera Research Center, Hoopes Vision, Draper, UT, USA; 4 McGovern Medical School at the University of Texas Health Science Center at Houston, Houston, TX, USA

**Keywords:** Cornea, Collagen, Cross-Linking Reagents, Riboflavin, Photosensitivity Disorders, Photophobia

## Abstract

In this case series, we report a potentially novel association of corneal collagen crosslinking (CCL) with the development of photophobia symptoms in a series of patients at a tertiary ophthalmology clinic and describe their clinical course. Photosensitivity is a rare and seemingly unpredictable complication of refractive surgery but can present as a disabling, bilateral ocular pain that requires immediate treatment. This complication, termed transient light-sensitivity syndrome (TLSS), can have a substantially delayed presentation after ocular procedures and is associated with inflammation of structures in the anterior chamber that can be imperceptible on slit-lamp examination. Traditionally, exposure to high-energy femtosecond lasers is hypothesized to create stromal gas bubbles powering postoperative inflammatory reactions. TLSS-like symptoms after CCL may be due to a secondary inflammatory response involving activated keratocytes and cytokine release. However, free radical damage from the interaction of riboflavin and ultraviolet in CCL may also drive this inflammatory process.

## INTRODUCTION

Unusual and unpredictable occurrences of photosensitivity without changes in visual acuity have been reported in several case reports described after refractive procedures, such as laser-assisted in situ keratomileusis (LASIK) and small incision lenticule extraction (SMILE). This complication, termed transient light-sensitivity syndrome (TLSS), is characterized by moderate-to-severe photophobia bilaterally that appears two to eight weeks after ocular procedures and is associated with inflammation of peripheral structures, such as the ciliary body, and possibly the trabecular meshwork and iris [1, 2]. TLSS typically does not present with clinical signs of inflammation or any other abnormalities of the anterior and posterior segments on slit-lamp exam [2, 3]. In our tertiary ophthalmology practice, we have observed several cases of acute TLSS-like symptoms weeks after corneal collagen crosslinking (CCL) procedures. CCL uses photopolymerization of riboflavin and ultraviolet-A radiation (UVA) to create inter- and intrafibrillar covalent bonds within the anterior stroma to slow the progression of keratoconus [4-7]. Typically, the epithelium is removed (EPI-Off) before CCL; however, transepithelial methods have also been used to preserve corneal thickness and reduce post-operative pain (EPI-On) [4, 8].

In this report, we describe the potential association of CCL and TLSS-like symptoms based on our clinical observations of patients who received CCL treatment. We also speculate possible mechanisms for this association.

## CASE DESCRIPTIONS

Similar to the phenomenon described after refractive procedures [2, 3, 9], we have observed a delayed-onset photosensitivity in seven of our patients who received CCL therapy with either the epithelium removed or kept in place. From 2014 to 2019, we have performed CCL in 125 patients using the Dresden protocol, delivering 3 milliwatts per square centimeter [mW/cm^2^] UVA for 30 min (dose = 5.4 joules per centimeter [J/cm]) with the FDA-approved KXL® system (Avedro, Waltham, MA). Photrexa viscous® (riboflavin 5’-phosphate in 20% dextran ophthalmic solution) 0.146% was applied topically on the eye for 30 min every two minutes prior to irradiation and then every two minutes for the 30-minute irradiation period. Postoperatively, all patients received topical moxifloxacin (Vigamox; Alcon; USA) 0.5% (four times daily for one week) and a standard regimen of topical prednisolone acetate 1% (four times daily for the first week, three times daily for the second week, two times daily for the third week, and once daily for the fourth week). 

A written informed consent was obtained from each patient prior to CCL. This case series was approved by the Ethics Board of Hoopes Durrie Rivera Research. 

Of our 125-patient sample, seven individuals experienced delayed photosensitivity symptoms similar to TLSS, yielding a 5.6% incidence over the last five years. Among the seven patients who experienced TLSS symptoms post-CCL, five were male, the majority were Caucasian, and age ranged from 14 to 54 years old. Additionally, all of these patients had stage 2 keratoconus by Amsler-Krumeich classification [10], prior to CCL treatment. More importantly, none of the seven patients had loss of corrected distance visual acuity (CDVA) after treatment of TLSS-like symptoms. Table 1 outlines our specific observations of these patients. Two patients who presented with typical TLSS symptoms after the CCL procedure are described in greater detail below.

**Table 1:** Observations of 7 patients with transient light-sensitivity syndrome (TLSS) symptoms post-CCL.

Patient No.	1	2	3	4	5	6	7
Age	17	28	54	14	16	43	40
Ethnicity/gender	Caucasian Male	Hispanic Male	Caucasian Female	Caucasian Male	Caucasian Male	Caucasian Female	Caucasian Male
Stage of Keratoconus	Stage 2	Stage 2	Stage 2	Stage 2	Stage 2	Stage 2	Stage 2
Involved eye	OD	OD	OS	OS	OS	OS	OD
Pre-CLL CDVA	20/40	20/150	20/25	20/25	20/30	20/25	20/25
Post-CCL CDVA	20/40	20/100	20/25	20/20	20/40	20/25	20/30
Post-TLSS	20/40	20/100	20/25	20/20	20/40	20/25	20/30
Kmax (diopter [D])	55	57	48	58	52	48	60
Pachymetry (micrometer [μm])	420	460	530	400	420	495	518
CCL Procedure	EPI-Off 3mW/cm^2^ x 30min	EPI-Off 3mW/cm^2^ x 30min	EPI-Off 3mW/cm^2^ x 30min	EPI-Off 3mW/cm^2^ x 30min	EPI-On 3mW/cm^2^ x 30min	EPI-On 3mW/cm^2^ x 30min	EPI-On 3mW/cm^2^ x 30min
Symptom onset post-CCL	7-wk	10-wk	6-wk	3-wk	6-wk	5-wk	9-wk
Severity scale*	1	1	2	1	2	1	1
Subjective dry eye	none	none	none	none	positive	Positive	none
Photosensitivity	positive	positive	positive	positive	positive	positive	positive
SLE	none	none	none	none	SPK	none	none
Corticosteroid administered	Fluorometholone	Fluorometholone	Fluorometholone	Fluorometholone	Fluorometholone	Fluorometholone	Fluorometholone
Symptom resolution after corticosteroid therapy	2-wk	2-wk	1-wk	6-wk	2-wk	1-wk	1-wk

CDVA: corrected distance visual acuity; CCL: corneal collagen cross-linking; Kmax: maximum keratometric reading; SLE: slit-lamp exam; SPK: superficial punctate keratitis; OD: right eye; OS: left eye; min: minute; wk: weeks

*Severity scale (0 = no pain, 1 = mild, 2 = moderate, 3 = severe). CCL method includes delivery of 3 milliwatts per square centimeter [mW/cm^2^] irradiance of UVA for 30 min (dose= 5.4 joules per centimeter [J/cm]); epithelium was removed for four patients (“EPI-Off”) and the epithelium was kept on for three patients (“EPI-On”). Fluorometholone (fluorometholone acetate 0.1%; Alcon; USA).

**Case 1**

A 54-year-old, Caucasian female presented to our clinic with stage 2 keratoconus left eye (OS) (Maximum keratometric reading [Kmax]=48 diopter [D], pachymetry=530 micrometer [μm]). She was a candidate for CCL and consented to undergo the procedure. The epithelial layer of the cornea OS was removed (EPI-Off) by debridement and CCL was performed with the Avedro KXL® system. After the procedure, a standard therapy of tapering corticosteroids was administered for four weeks. There were no intraoperative complications. Before CCL, her CDVA was 20/25, and after CCL, her CDVA was 20/25 as well. Six weeks post-CCL, she acutely presented to the clinic with moderate photosensitivity OS, no subjective dry eye, and with no significant findings on slit-lamp exam. Her sensitivity to light resolved after one week of fluorometholone (fluorometholone acetate 0.1%; Alcon; USA) therapy four times a day. After treatment of TLSS-like symptoms, her CDVA remained 20/25 and there was no loss of visual acuity. 

**Case 2**

A 40-year-old, Caucasian male presented to us with stage 2 keratoconus right eye (OD) (Kmax=60 D, pachymetry=518 μm) and opted to proceed with CCL. The procedure was uneventful with no intraoperative complications. The epithelial layer remained intact (EPI-On), and CCL was performed with the Avedro KXL® system. He was prescribed a standard corticosteroid regimen tapered over four weeks. Before CCL, his CDVA was 20/25, and after CCL, his CDVA was 20/30. Nine weeks post-CCL, he presented to the clinic with a one-day history of mild symptoms of photophobia OD but denied dry eye symptoms. On slit-lamp examination, external and anterior segments were normal OD. He was prescribed fluorometholone (fluorometholone acetate 0.1%; Alcon; USA) four times a day and his symptoms were alleviated within one week. After his symptoms of TLSS were relieved, his CDVA did not change and presented as 20/30.

**Summary of Clinical Cases**

Among our seven patients with photophobia symptoms post-CCL, four had their epithelial layer removed prior to CCL, and the other three patients underwent the transepithelial method. Symptoms of TLSS acutely emerged within a range of five weeks to three months post-CCL. All seven patients reported subjective photosensitivity that varied in severity, although most cases were mild to moderate photophobia. Two patients complained of subjective dry eye. All of these patients also had no elevations in intraocular pressure (IOP). Slit-lamp exam was unremarkable for signs of inflammation in the anterior chamber (AC) in six cases, and one patient had endothelial inflammation on exam. The patient was a 16-year-old Caucasian male who presented with minimal superficial punctate keratopathy (SPK) and moderate photosensitivity OS six-week post-CCL. All seven patients were given fluorometholone (fluorometholone acetate 0.1%; Alcon; USA) with subsequent relief of symptoms within one to six weeks. More importantly, none of the seven patients had loss of CDVA after treatment of TLSS-like symptoms (Table 1).

## DISCUSSION

In our patient population, seven patients experienced delayed photophobia, similar to symptoms seen in TLSS, after undergoing CCL. No definite etiologies are accepted for the development of TLSS; however, many theories have been proposed giving insight into how the severe photophobia symptoms develop in LASIK and SMILE [1-3, 9]. These propositions describe an underlying inflammatory mechanism that may occur in other ocular surface procedures, such as in corneal crosslinking treatment for keratoconus in our patient population.

Several studies have reported symptoms of extreme light sensitivity after femtosecond flap creation performed with LASIK. Stonecipher et al. described a 1% incidence of TLSS among the patients who received LASIK with femtosecond laser flap creation in their study, especially among those who received high initial energy from the laser [9]. Similarly, the incidence of TLSS among patients who also underwent LASIK using a femtosecond laser was 1.3% in a study [3]. Both studies described the bilateral photophobia with normal uncorrected distance visual acuity (UDVA) with no detectable signs of inflammation in the AC, and all patients’ rapidly responded to topical corticosteroids, such as dexamethasone eye drops. When treated with aggressive topical corticosteroid therapy, TLSS symptoms typically resolve within a few days or weeks [3, 9]. Cyclosporine ophthalmic emulsion 0.05% (Restasis®, Allergan, Irvine, CA, USA) and punctal plugs may also be helpful to relieve the photophobia symptoms [9].

The apparent efficacy of these immunosuppressant agents indicates that the etiology of TLSS is likely an inflammatory process. Shock-wave exposure to keratocytes and corneal nerves with high laser pulse energy is a likely cause of the local activation of keratocytes [1, 9]. The presence of activated keratocytes at the stromal-corneal flap interface seen with confocal microscopy in 30% of TLSS patients in their study [3]. Perhaps the inflammatory process involving the activated keratocytes causes secondary pain and photophobia in patients who underwent CCL.

Although procedurally different, the structures secondarily inflamed in LASIK may be similar to those affected in CCL. After femtosecond flap formation, peripheral displacement of bubbles created in the stromal interface irritates the ciliary body. Resulting cytokines migrate to the intersection of the sclera, limbus, and iris, producing an inflammatory response [3, 9]. Within the techniques used for flap formation in LASIK, TLSS is identified as a complication specific to the use of femtosecond lasers, especially with the use of high energies, compared to the use of microkeratome [1, 3, 11]. Moreover, TLSS has been reported in patients with clinical haze after receiving high laser energy exposure during LASIK possibly due to subsequent keratocyte activation [12]. The occurrence of TLSS after SMILE was described and proposed that the development of delayed-onset extreme photophobia postoperatively was probably due to inflammatory responses, involving cytokines and activated keratocytes, beyond the disruption of tissues and neural connections from lenticule extraction alone [2]. Etiology of TLSS is not specific to SMILE or LASIK, as inflammation of common nearby structures is a common unifying theme. We believe that inflammation of these same structures may underlie the photosensitivity seen in our patients after CCL.

Our first theory of the development of delayed-onset photophobia in CCL patients involves a cytokine-mediated, inflammatory mechanism. CCL results in lacunar edema and dose-dependent damage to keratocytes in the anterior stroma [13-15]. Three to six months after a CCL procedure, stromal healing is noted with an increase in the density of the extracellular matrix (ECM) accompanied by an increased number of activated keratocyte nuclei involved in stromal wound healing [4, 16]. Corneal haze in the stroma has been reported and this complication may be due to the activated keratocytes that proliferate in the stroma two months after CCL [17-19]. Secondary inflammation and stromal wound healing continue through 36 months after CCL [16]. These findings indicate that secondary inflammation may cause the delayed-onset photophobia symptoms by an increase in activated keratocytes involved in stromal healing.

**Figure 1:**
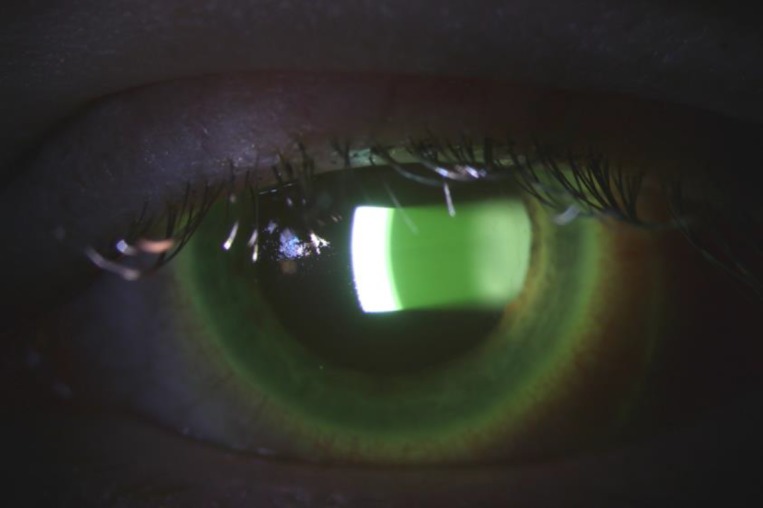
A typical slit-lamp picture after collagen cross-linking (CCL), indicating presence of riboflavin in the anterior chamber. Courtesy of Dr. Majid Moshirfar

**Figure 2:**
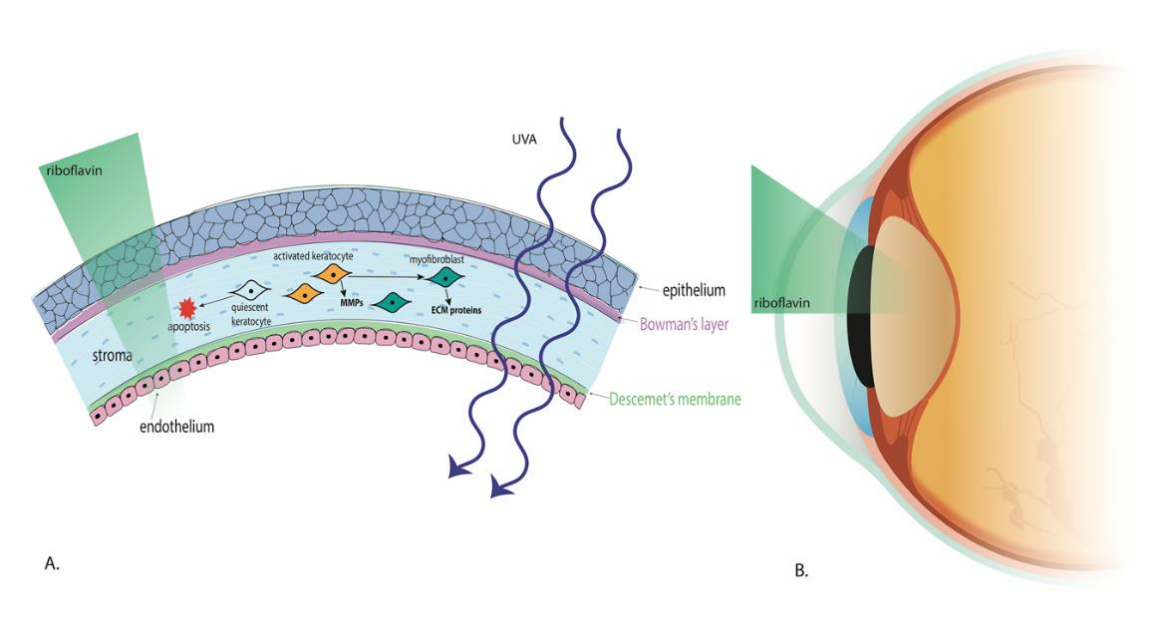
Two speculated mechanisms of the development of transient light-sensitivity syndrome like symptoms in patients after collagen cross-linking (CCL). Figure A indicates a cytokine-mediated process involving activated keratocytes driving inflammation. Figure B indicates the proposed penetration of riboflavin into the anterior chamber of the eye during CCL. If riboflavin is excited by UVA light, this may possibly irritate uveal structures and produce secondary pain

Another idea that possibly explains the TLSS-like symptoms in patients post-CCL may be direct photosensitivity and aggravation of peripheral structures by riboflavin excitation in the AC. If riboflavin can penetrate through deeper layers of the cornea into the AC, there is a theoretical risk of phototoxicity to the iris, anterior lens, and other structures of the anterior segment (Fig. 1) When riboflavin is irradiated with UVA, reactive oxygen species are created that can cause cellular damage. For example, treatment of riboflavin with a standard 3 mW/cm^2^ irradiance of UVA has been shown to damage the endothelial layer and limbal epithelial stem cells in thin corneas [20-22]. Endotheliitis has been reported in several cases after CCL procedures, which further suggests damage to the endothelial layer is possible [23, 24]. The topical application of 0.1% riboflavin to the cornea resulted in a 0.015% concentration of riboflavin in the endothelium, which suggests that riboflavin may diffuse through the cornea into the AC [25]. Additionally, some studies indicate the possibility of retinal phototoxicity due to UVA radiation during corneal crosslinking [26, 27]. When riboflavin and UVA are applied to a localized area of the porcine cornea, the stiffening effect of CCL extends beyond the region in which the riboflavin and UVA were applied [28]. Fig. 2 depicts two theories, an inflammatory mechanism and direct phototoxicity of irradiated riboflavin in the AC. We speculate that these two interdependent mechanisms may be associated with TLSS-like symptoms in patient’s post-CCL.

Limitations of our observational study include the small sample size of patients with photophobia symptoms observed after CCL procedures at our tertiary ophthalmology clinic, possibly resulting in low statistical power of our study. Additionally, since all of our seven patients observed were treated for stage 2 keratoconus with CCL procedures, confounding variables may be introduced into our study. Another limitation in our methods is that inflammation was determined only by slit-lamp exam and was not precisely quantified; confocal microscopy and ocular fluorometry were not used to examine inflammation and flare in our seven patients. A strength of our study is that we are the first group to report this clinical observation of transient photophobia after CCL procedures. We have observed that TLSS-like symptoms occur even with CCL procedures involving the epithelium intact or removed. Therefore, these postoperative symptoms were not exclusively limited to refractive procedures.

## CONCLUSION

Symptoms of TLSS are uncommon, but moderately painful, and may be associated with CCL procedures. The incidence of TLSS-like symptoms in our patients described may be due to a secondary inflammatory response involving activated keratocytes and cytokine release during tissue remodeling and healing. These symptoms may also be associated with possible phototoxicity from free radical damage from riboflavin and UVA extending into the AC. Further research should be conducted on the wound healing process and which structures are involved in the inflammatory process in CCL procedures in light of the appearance of extremely delayed photophobia after treatment, possibly due to secondary inflammation. Additionally, more studies should be conducted determining the extent of riboflavin penetration into the AC, and the factors that may increase the risk of phototoxicity of riboflavin with excitation from UVA. There is a possibility that TLSS symptoms can occur when using irradiance higher than 3 mW/cm^2^ UVA with shorter procedure times. Additionally, TLSS symptoms may increase if no corticosteroids were immediately prescribed postoperatively. The use of corticosteroids after CCL, while routine for four weeks, may be reintroduced when symptoms of TLSS appear.

## DISCLOSURE

Ethical issues have been completely observed by the authors. All named authors meet the International Committee of Medical Journal Editors (ICMJE) criteria for authorship of this manuscript, take responsibility for the integrity of the work as a whole, and have given final approval for the version to be published. No conflict of interest has been presented.

## Funding/Support:

Research to Prevent Blindness, New York, USA.
